# Supplementary material for: Effects of Trichoderma harzianum combined with Phanerochaete chrysosporium on lignin degradation and humification during chicken manure and rice husk composting
Source: Front Microbiol. 2025 Feb 28;16:1515931. doi: 10.3389/fmicb.2025.1515931 (PMC11906335; doi:10.3389/fmicb.2025.1515931)
Supplement: Supplementary file 1 [file Data_Sheet_1.zip › 6.Function/CK0_HS/FunGuild/l1/CK0_vs_HS_STAMP.pdf]

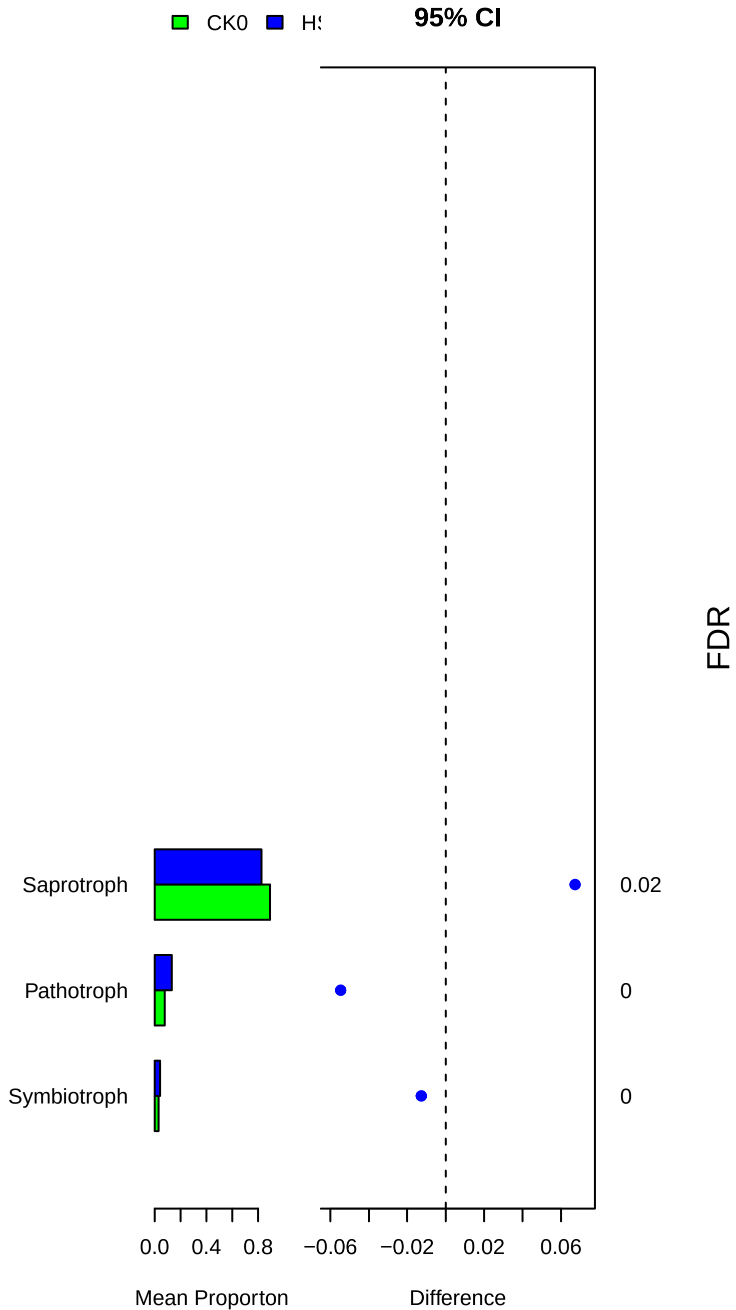
CK0
HS
95% CI
FDR
Saprotroph
Pathotroph
Symbiotroph
0.02
0
0
0.0
0.4
0.8
−0.06
−0.02
0.02
0.06
Mean Proporton
Difference
